# Supplementary material for: Machine learning prediction and tau-based screening identifies potential Alzheimer’s disease genes relevant to immunity
Source: Commun Biol. 2022 Feb 11;5:125. doi: 10.1038/s42003-022-03068-7 (PMC8837797; doi:10.1038/s42003-022-03068-7)
Supplement: Supplementary file 2 — Supplementary Information [file 42003_2022_3068_MOESM2_ESM.pdf]

SUPPLEMENTARY INFORMATION FOR COMMSBIO-20-3222R1

Machine learning prediction and tau-based screening identifies potential Alzheimer's disease genes relevant to immunity

Jessica Binder[1], Oleg Ursu[1^], Cristian Bologa[1], Shanya Jiang[2], Nicole Maphis[2], Somayeh Dadras[2], Devon Chisholm[2], Jason Weick[3], Orrin Myers[1], Praveen Kumar[1], Jeremy J. Yang[1], Kiran Bhaskar[2,4], and Tudor I. Oprea[1,5,6].

1. Department of Internal Medicine, University of New Mexico School of Medicine, Albuquerque, NM 87131, USA
2. Department of Molecular Genetics and Microbiology, University of New Mexico School of Medicine, Albuquerque, NM 87131, USA
3. Department of Neuroscience, University of New Mexico School of Medicine, Albuquerque, NM 87131, USA
4. Department of Neurology, University of New Mexico School of Medicine, Albuquerque, NM 87131, USA
5. Department of Rheumatology and Inflammation Research, Institute of Medicine, Sahlgrenska Academy at Gothenburg University, 40530 Gothenburg, Sweden.
6. Novo Nordisk Foundation Center for Protein Research, Faculty of Health and Medical Sciences, University of Copenhagen, 2200 Copenhagen, Denmark.

- Supplementary Table 1
- Supplementary Table 2
- Supplementary Table 3
- Supplementary Table 4
- Supplementary Table 5
- Supplementary Table 6
- Supplementary Table 7
- Supplementary Table 8
- Supplementary Table 9
- Supplementary Table 10
- Supplementary Figure 1
- Supplementary Figure 2
- Supplementary Figure 3
- Supplementary Figure 4
- Supplementary Figure 5
- Supplementary Figure 6
- Supplementary Figure 7
- Supplementary Figure 8
- Supplementary Figure 9
- Supplementary Figure 10
- Supplementary Figure 11
- Supplementary Figure 12
- Supplementary Note 1
- Supplementary References

SUPPLEMENTARY TABLES

Supplementary Table 1. Inventory of AD protein knowledge graph data sources. *(Note, number of attributes reflect the February 2018 release of each database/resource)*

**Data type**	**Resource**	**Attributes**
Cellular pathways	Reactome[1]	303,681
Cellular pathways	KEGG[2]	27,683
Gene expression	CCLE[5]	19,006,134
Gene expression	GTEx[3]	2,612,227
Gene expression	HPA[4]	949,199
Gene function	GO[5]	145,824
Protein classification	InterPro[6]	467,163
Gene perturbation signatures	LINCS[7]	230,111,315
Mouse phenotype	IMPC[8]	2,153,999
Rat phenotype	RGD[9]	117,606
Human disease/phenotype	ClinVar[10]	881,357
Human disease/phenotype	OMIM[11]	5,365
Protein-protein interactions	STRING[12]	5,080,023

Supplementary Table 2: List of positively associated AD genes used for ML training ("train") and testing ("test", shaded).

Training				Test	
**UniProt ID**	**HGCN symbol**	**UniProt ID**	**HGCN symbol**	**UniProt ID**	**HGCN symbol**
Q8IZH2	XRN1	P01584	IL1B	P01023	A2M
P35348	ADRA1A	P05231	IL6	P50406	HTR6
P05091	ALDH2	P05019	IGF1	Q16643	DBN1
Q92870	APBB2	P22301	IL10	Q06481	APLP2
P30556	AGTR1	P60568	IL2	O00213	APBB1
P03950	ANG	P45983	MAPK8	P51693	APLP1
P30533	LRPAP1	O00408	PDE2A	P06241	FYN
Q16853	AOC3	P27986	PIK3R1	P78352	DLG4
P02647	APOA1	O14920	IKBKB	Q9H2W1	MS4A6A
Q13315	ATM	P06213	INSR	P14735	IDE
Q92934	BAD	O60674	JAK2	Q16555	DPYSL2
P23560	BDNF	Q07866	KLC1	P01011	SERPINA3
P32246	CCR1	Q12791	KCNMA1	P10997	IAPP
Q92793	CREBBP	Q9UEF7	KL	Q9Y6A2	CYP46A1
P06276	BCHE	P14151	SELL	Q8IU99	CALHM1
O15516	CLOCK	P16109	SELP	P42261	GRIA1
P01034	CST3	O15151	MDM4	Q96BI3	APH1A
P01588	EPO	Q9NPG2	NGB	O96008	TOMM40
P00533	EGFR	Q96SB3	PPP1R9B	P56817	BACE1
Q92731	ESR2	Q16236	NFE2L2	Q9UMX0	UBQLN1
O14556	GAPDHS	P30086	PEBP1	P27338	MAOB
P04406	GAPDH	P11086	PNMT	P17927	CR1
P30711	GSTT1	O60260	PRKN	Q9Y5Z0	BACE2
P49840	GSK3A	P01286	GHRH		
P09211	GSTP1	Q6ZVD7	STOX1		
P0DMV8	HSPA1A	Q03519	TAP2		
P0DMV9	HSPA1B				

Supplementary Table 3. Confusion matrix for the weighted (clear) and balanced (shaded) MPxgb AD models

**Test Set**		Predicted			
		Pos	Neg	Pos	Neg
	Positive	16	7	**20**	3
Actual	Negative	94	106	41	**159**

Supplementary Table 4. Details of human brain samples used, all collected from temporal cortices. We obtained tissue samples from Northwestern Cognitive Neurology & Alzheimer's Disease Center (CNADC) Neuropathology Core in which consent was provided to the tissue repository. Please see https://www.brain.northwestern.edu/join/brain-donation.html

Sample ID	PT ID	Age	Sex	Aut #	Clin Dx	Path Dx 1	Path Dx 2	Path Dx 3
Ctrl1	852	76	F	A13-75	NCI	AD-aging (0, II, 0)	Mild vascular disease	
Ctrl2	365	75	F	A07-37	NCI	AD-aging (0, II, N/C)	Bilateral organizing SDH	Alzheimer type II astrocytes
Ctrl3	6247	35	M	A03-06	NC-DM, Htn, sarcoid, CAD	NC (0, 0, N/A), edema	Arteriolosclerosis	Dentate nuc gliosis
Ctrl4	6358	88	M	A07-151	NC (CLL, CHF)	AD-aging (C, II, N/C)		
Ctrl5	421	82	F	A18-148	NCI Super Ager	AD-aging (not AD - A0, B1, C0)	Mild vascular disease	Incidental LBs, nigra & locus
Ctrl6	562	81	M	A16-285	LBD	AD-aging (ADNC low - A3, B1, C2)	mild vascular disease	remote microinfarct, hippo
Ctrl7	1033	87	F	A19-50	NCI	AD-aging (ADNC low - A3, B1, C3)	moderate vascular disease	
Ctrl8	6379	95	M	A10-04	Stroke(?) POAD	AD-aging (0, I, N/C)	Multiple infarcts	Vascular disease
AD1	252	92	F	AX11-124	PRAD	AD (C, V, high)	Vascular disease, severe	
AD2	510	79	F	A09-140	PRAD	AD (C, VI, high)		
AD3	553	78	M	A10-140	PRAD	AD (C, VI, high)		
AD4	872	67	F	A10-114	PRAD	AD (C, VI, high)		
AD5	1415	83	F	A10-143	PRAD	AD (C, VI, high)	Vascular disease	Multiple microinfarcts
AD6	1429	65	M	AX11-109	CBS	AD (C, VI, high)		
AD7	159	71	M	A11-46	PRAD	AD (C, VI, high)	mild vascular disease	AGD
AD8	1066	82	M	A12-28	PPA	AD (ADNC high - A3, B3, C3)	vascular disease, severe, large IF	AGD

(Ctrl – Control; AD – Alzheimer's disease; M/F – Males/Females; NCI – No cognitive impairment; NC – normal controls; CLL - Chronic Lymphocytic Leukemia; CHF – Congestive heart failure; PRAD – Probable Alzheimer's disease; CBS – Corticobasal syndrome; 0, I, II, V and VI – Braak Staging; SDH - Subdural hematomas)

Supplementary Table 5. A list of the bottom 10 genes predicted from the MPxgb(AD) model. The Predicted probability column is the XGboost classifier probability that a particular gene belongs to the "AD positive" class.

UniProt ID	HGCN symbol	Predicted probability
O14901	KLF11	0.000137
Q9Y6J8	STYXL1	0.000135
Q6NUN0	ACSM5	0.000134
Q9NY57	STK32B	0.000133
O60911	CTSV	0.000131
O43933	PEX1	0.000130
P98082	DAB2	0.000125
P09017	HOXC4	0.000115
Q13825	AUH	0.000111
O60825	PFKFB2	0.000111

Supplementary Table 6: metapaths are implemented via SQL queries which extract matching metapath entities from TCRD to build an in-memory knowledge graph

Metapath	SQL
Target – [member of] -> PPI (protein-protein interaction network) <- [member of] – Protein – [associated with] -> Disease	SELECT protein1_id, protein2_id, score AS combined_score FROM ppi WHERE ppitype = 'STRINGDB'
Target – [member of] -> Pathway (KEGG) <- [member of] – Protein – [associated with] -> Disease	SELECT protein_id, SUBSTR(id_in_source, 6) AS kegg_pathway_id FROM pathway WHERE pwtype = 'KEGG'
Target – [member of] -> Pathway (Reactome) <- [member of] – Protein – [associated with] -> Disease	SELECT protein_id, id_in_source AS reactome_id, name AS evidence FROM pathway WHERE pwtype = 'Reactome'
Target – [member of] -> Family (InterPro) <- [member of] – Protein – [associated with] -> Disease	SELECT DISTINCT protein_id, value AS entry_ac FROM xref WHERE xtype = 'InterPro'
Target – [member of] -> Family (Pfam) <- [member of] – Protein – [associated with] -> Disease	SELECT DISTINCT protein_id, value AS entry_ac FROM xref WHERE xtype = 'Pfam'
Target – [member of] -> GO Term <- [member of] – Protein – [associated with] -> Disease	SELECT DISTINCT protein_id, value AS entry_ac FROM xref WHERE xtype = 'PROSITE'
Target – [member of] -> Family (PROSITE) <- [member of] – Protein – [associated with] -> Disease	SELECT protein_id, go_id FROM goa

Supplementary Table 7: In addition to the meta-path based features, a set of static features is generated for sources invariant with disease query: GTEx, LINCS, CCLE and HPA.

Static-feature	SQL
GTEx gene expression, tissue-specific	SELECT protein_id, CAST(AVG(tpm) AS DECIMAL(5,3)) AS median_tpm, tissue AS tissue_type_detail FROM gtex
Cancer Cell-line Encyclopedia (CCLE)	SELECT protein_id, cell_id, tissue, number_value AS expression FROM expression WHERE etype = 'ccle'
Library of Network-base Cell Signatures (LINCS), cell-line specific	SELECT protein_id, CONCAT(pert_dcid, ':', cellid) AS col_id, zscore FROM lincs
Human Protein Atlas (HPA) expression, tissue-specific	SELECT DISTINCT protein_id, tissue AS col_id, qual_value AS level FROM expression WHERE etype = 'HPA'

Supplementary Table 8. List and source of siRNA used for individual gene knockdowns

siRNA Target	Company and Catalog #	siRNA ID
AKNA	Thermofisher# 4427037	s37301
BCO2	Thermofisher# 4427037	s38257
CCNY	Thermofisher# 4427037	s47720
CRTAM	Thermofisher# 4427037	s32084
FAM92B	Thermofisher# 4427037	s50460
FOXP4	Thermofisher# 4427037	s41930
FRRS1	Thermofisher# 4427037	s52913
GRIN2C	Thermofisher# 4427037	s6178
IL17REL	Thermofisher# 4427037	s53410
LILRA3	Thermofisher# 4427037	s21724
LMO4	Thermofisher# 4427037	s16258
NDRG2	Thermofisher# 4427037	s33034
PIBF1	Thermofisher# 4427037	s20482
RAB40A	Thermofisher# 4427037	s44481
SCGB3A1	Thermofisher# 4427037	s195566
SLC44A2	Thermofisher# 4427037	s32793
SPOP	Thermofisher# 4427037	s15954
STARD3	Thermofisher# 4427037	s21541
TMEFF2	Thermofisher# 4427037	s24304
TXNDC12	Thermofisher# 4427037	s27322
scramble	Thermofisher# 4427037	

Supplementary Table 9. List of TaqMan assays used

Gene Name	Gene Symbol	Assay ID
AT-Hook Transcription Factor	*AKNA*	Hs00980996_m1
Beta-Carotene Oxygenase 2	*BCO2*	Hs00230564_m1
Cyclin Y	*CCNY*	Hs01554033_m1
Cytotoxic And Regulatory T Cell Molecule	*CRTAM*	Hs00219699_m1
Family With Sequence Similarity 92 Member B	*FAM92B*	Hs00420415_m1
Forkhead Box P4	*FOXP4*	Hs01055269_m1
Ferric Chelate Reductase 1	*FRRS1*	Hs01395066_m1
Glutamate Ionotropic Receptor NMDA Type Subunit 2C	*GRIN2C*	Hs01016628_m1
Interleukin 17 Receptor E Like	*IL17REL*	Hs00913980_m1
Leukocyte Immunoglobulin Like Receptor A3	*LILRA3*	Hs00846590_s1
LIM Domain Only 4	*LM04*	Hs01086790_m1
N-myc DownrRgulated Gene Family Member 2	*NDRG2*	Hs01045116_g1
Progesterone Immunomodulatory Binding Factor 1	*PIBF1*	Hs00197131_m1
Member RAS Oncogene Family gene encodes a member of the Rab40 subfamily of Rab small GTP-binding proteins that contains a C-terminal suppressors of cytokine signaling box	*RAB40A*	Hs05575393_g1
Secretoglobin Family 3A Member 1	*SCGB3A1*	Hs00369360_g1
Solute Carrier Family 44 Member 2	*SLC44A2*	Hs01105936_m1
Speckle Type BTB/POZ Protein	*SPOP*	Hs00737433_m1
StAR Related Lipid Transfer Domain Containing 3	*STARD3*	Hs00199052_m1
Transmembrane Protein with EGF Like And Two Follistatin Like Domains 2	*TMEFF2*	Hs01086902_m1
Thioredoxin Domain Containing 12	*TXNDC12*	Hs00210841_m1
Peroxisomal Biogenesis Factor 1	*PEX1*	Hs00166599_m1
DAB Adaptor Protein 2	*DAB2*	Hs01122246_m1
Cathepsin V	*CTSV*	Hs00952036_m1
Homeobox C4	*HOXC4*	Hs00538088_m1
AU RNA Binding Methylglutaconyl-CoA Hydratase	*AUH*	Hs01060457_g1
6-Phosphofructo-2-Kinase/Fructose-2,6-Bisphosphatase 2	*PFKFB2*	Hs01015408_m1
Kruppel Like Factor 11	*KLF11*	Hs00231614_m1
Serine/Threonine/Tyrosine Interacting Like 1	*STYXL1*	Hs01553273_m1
Acyl-CoA Synthetase Medium Chain Family Member 5	*ACSM5*	Hs00384982_m1
Serine/Threonine Kinase 32B	*STK32B*	Hs01031449_m1
Euk 18S rRNA	*Euk 18S rRNA*	4319413E

Supplementary Table 10. Antibodies and the sources used for Western blot and Immunohistochemical analysis

Antibody	Species	Company and Catalog #	Dilutions
AKNA	Rabbit	Abcam#ab220392	1:1000 (WB) 1:500 (IF)
BCO2	Rabbit	Thermofisher#PA5-24527	1:1000 (WB) 1:250 (IF)
CCNY	Rabbit	Thermofisher#PA5-23644	1:1000 (WB) 1:250 (IF)
CRTAM	Mouse	SantaCruz# sc-390581	1:1000 (WB) 1:500 (IF)
FAM92B	Rabbit	Thermofisher#PA5-59398	1:1000 (WB) 1:250 (IF)
FOXP4	Rabbit	Thermofisher#PA5-49682	1:1000 (WB) 1:250 (IF)
FRRS1	Rabbit	Abcam# ab121538	1:1000 (WB) 1:500 (IF)
GRIN2C	Rabbit	Thermofisher#OPA1-04020	1:1000 (WB) 1:250 (IF)
IL17REL	Rabbit	Abcam# ab126399	1:1000 (WB) 1:500 (IF)
LILRA3	Rabbit	Thermofisher# PA5-28902	1:1000 (WB) 1:250 (IF)
LMO4	Rabbit	Thermofisher# PA5-24248	1:1000 (WB) 1:250 (IF)
NDRG2	Mouse	SantaCruz# sc-376202	1:1000 (WB) 1:500 (IF)
PIBF1	Rabbit	Thermofisher# PA5-34514	1:1000 (WB) 1:500 (IF)
RAB40A	Rabbit	Thermofisher# PA5-69848	1:1000 (WB) 1:250 (IF)
SCGB3A1	Mouse	Abcam# ab201604	1:1000 (WB) 1:500 (IF)
SLC44A2	Rabbit	Thermofisher# PA5-67127	1:1000 (WB) 1:500 (IF)
SPOP	Rabbit	Thermofisher# PA5-28522	1:1000 (WB) 1:250 (IF)
STARD3	Rabbit	Thermofisher#PA1-562	1:1000 (WB) 1:250 (IF)
TMEFF2	Rabbit	Thermofisher#PA5-53327	1:1000 (WB) 1:250 (IF)
TXNDC12	Rabbit	Thermofisher#PA5-24798	1:1000 (WB) 1:250 (IF)
CTSV	rabbit	Abcam#ab166894	1:1000 (WB)
DAB2	rabbit	Abcam#ab33441	1:1000 (WB)
HOXC4	rabbit	Abcam#ab76093	1:1000 (WB)
AUH	rabbit	Abcam#ab157453	1:1000 (WB)
PFKFB2	rabbit	Abcam#ab234865	1:1000 (WB)
KLF11	mouse	Novus Biologicals#H00008462-M03	1:1000 (WB)
STYXL1	mouse	Novus Biologicals#H00051657-B02P	1:1000 (WB)
ACSM5	mouse	Novus Biologicals#NBP2-01874	1:1000 (WB)
STK32B	rabbit	Novus Biologicals#NBP1-32343	1:1000 (WB)
PEX1	rabbit	Thermo Scientific#13669-1-AP	1:1000 (WB)
Beta-actin	Mouse	Abcam#ab8226	1:10,000 (WB)
AT180	Mouse	Thermo Scientific, MN1040	1:5000 (WB)
AT8	Mouse	Thermo Scientific, MN1020	1:10,000 (WB)
GAPDH	Mouse	Millipore, CB1001-500UG	1:20,000 (WB)
Tau12	Mouse	Abcam, ab74137 Millipore, MAB2241	1:20,000 (WB)

SUPPLEMENTARY FIGURES

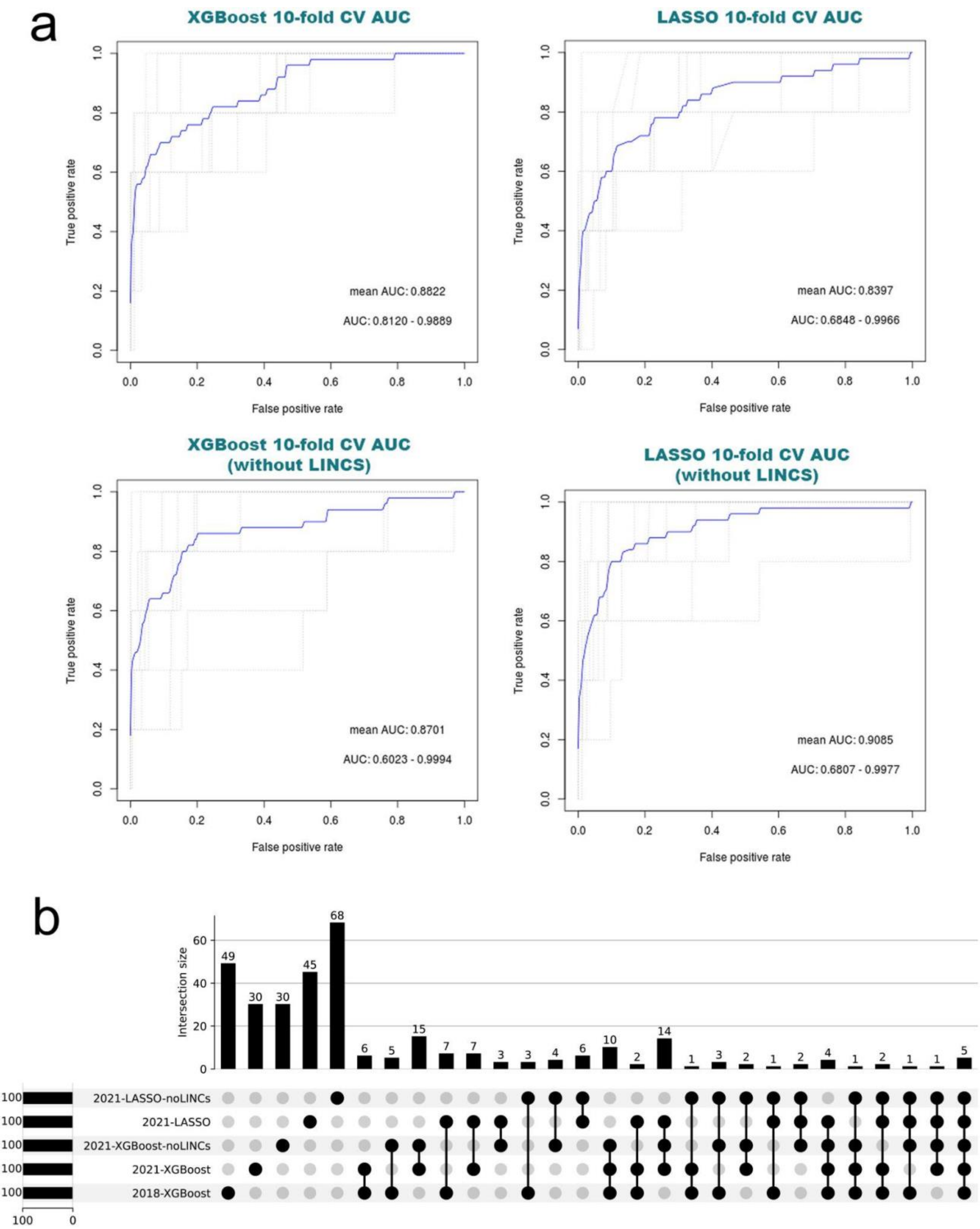

Supplementary Figure 1. Comparison of different cross validation models. a) AUC-ROC curves of XGBoost and LASSO with or without LINCS selected. Dashed lines represent AUC-ROC values in 10-fold whereas the bold blue line represents the mean of

those 10-fold AUC-ROC values. For XGBoost with LINCS, the value of AUC-ROC ranged from 0.8120-0.9889 (mean: 0.8822) in the ten-fold CV. For LASSO regression to compare the performance of classifiers, the value of AUC-ROC for LASSO with LINCS models varied from 0.6848-0.9966 (mean: 0.8397) in the 10-fold CV. In comparison to XGBoost models, LASSO models have a considerable variance in AUC-ROC. For XGBoost without LINCS, the value of AUC-ROC ranged from 0.6023-0.9994 (mean: 0.8701) in the ten-fold CV. For LASSO without LINCS, the value of AUC-ROC ranged from 0.6807-0.9977 (mean: 0.9085) in the ten-fold CV. Overall with LINCS, XGBoost performs slightly better, and LASSO performs slightly better without LINCS. **b)** Using the "Compare Sets Appyter" [63], we show the intersection between the 2018 and 2021 models for the top 100 predicted genes. For the 2018 model, 49 genes are included in at least one of the 2021 models, whereas the 2021 LASSO-noLINCS model has the most unique gene lists.

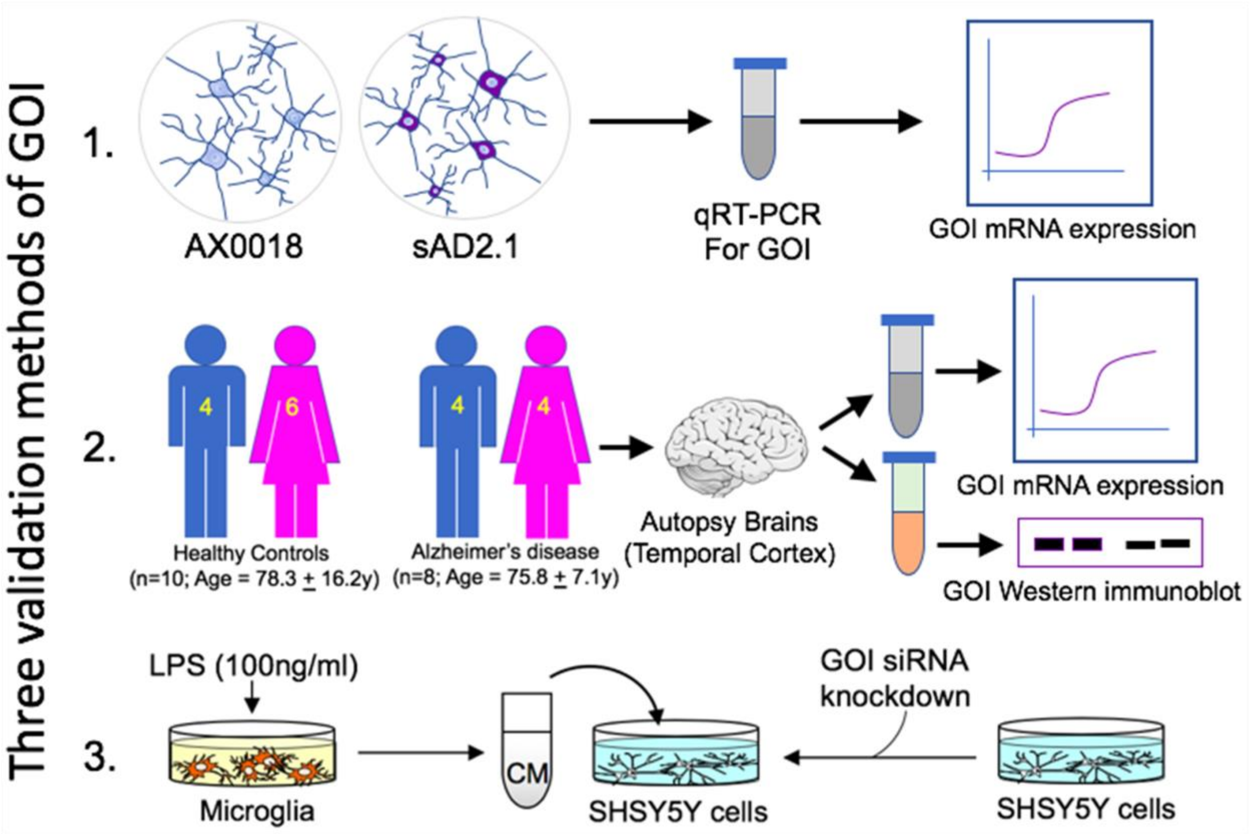

Supplementary Figure 2. Schematic showing the study design and overview of the data for the validation of predicted genes. A three-prong validation pipeline designed to validate the association of predicted genes with AD-related tau phenotype. The first set of validations investigate the mRNA levels of genes of interest (GOI; all twenty predicted genes) in human inducible pluripotent stem cell derived neurons (iPSNs) from sporadic AD (sAD2.1 – from Coriell Institute; # GM24666; 83 years old male) and control (AX0018 – from Axol Bioscience; #ax0018-kit; 74 years old male). The second set of validations involve mRNA and proteins analysis of each GOI from clinically diagnosed post-mortem brain samples (temporal cortices) from sporadic AD and non-demented healthy controls. The third set of validations include investigation of inflammation-induced tau phosphorylation following siRNA-mediated knockdown of GOI in SH-SY5Y human neuroblastoma cells. In this model, first the GOI is knocked down by siRNA followed by treating them with conditioned media (CM) derived from LPS (100 ng/ml)-activated microglia. Levels of phospho(p)-Ser199/pS202 (AT8 site) and pT231 (AT180 site) are assessed in SH-SY5Y cells.

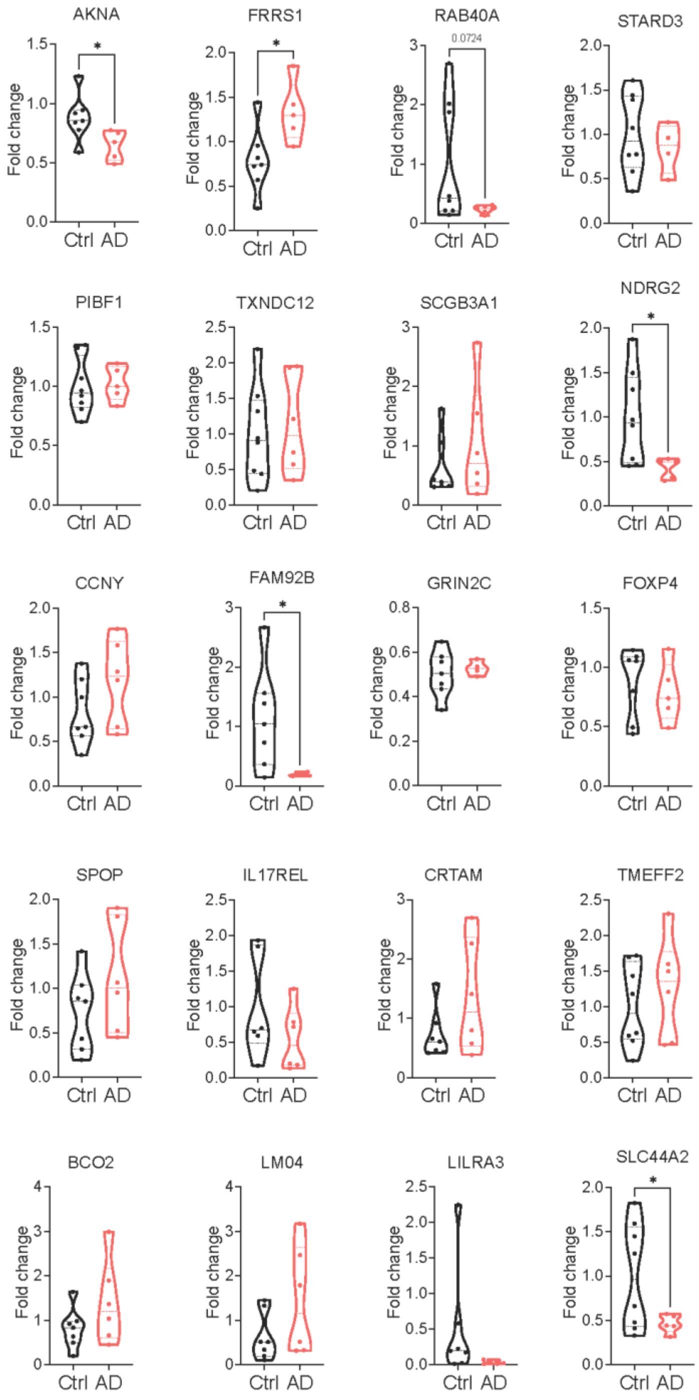

Supplementary Figure 3. Violin plots showing mRNA level of AKNA, FRRS1, NDRG2, FAM92B, and SLC44A2 are significantly altered in the post-mortem autopsy brains with Alzheimer's disease (AD). qRT-PCR analysis showing mRNA levels of AKNA, NDRG2, FAM92B, and SLC44A2 significantly down-regulated, but FRRS1 is significantly up-regulated in post-mortem temporal cortices of AD compared to age matched controls (Ctrl). Data are expressed as mean ± SEM; n=10 healthy controls and n=8 sporadic AD; n=3 technical replicates; *$p<0.05$; two-tailed *t* test, welch-corrected, and Tukey's for outlier removal. P values for AKNA = 0.0314, FRRS1 = 0.0254, NDRG2 = 0.0172, FAM92B = 0.0430, and SLC44A2 = 0.0384.

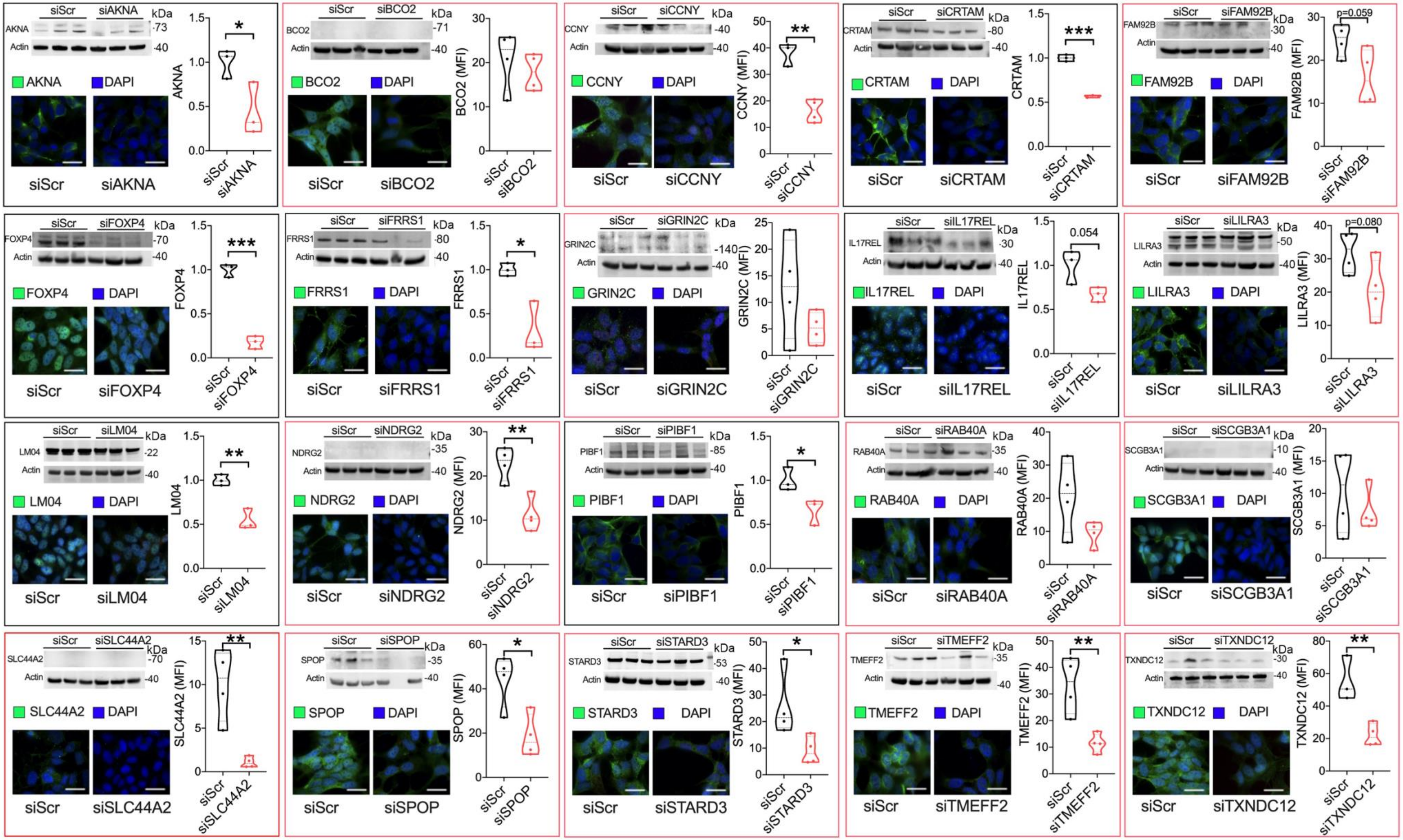

Supplementary Figure 4: siRNA mediated knockdown of predicted GOI. Undifferentiated SH-SY5Y cells were treated with siRNAs specific to predicted GOI 24 h prior to western blot and immunofluorescence analysis for the target proteins. Gene knockdowns were validated by quantifying Western blots (black squares) or by quantitative immunofluorescence (for the ones couldn't be confirmed by Western blot knockdowns (red squares) using scoring for the immunoreactive areas in four random fields and determining mean fluorescence intensity (MFI) by Image J. Scale bar: 20 µm. Note some genes didn't show either expression or responded to knockdowns (e.g. BCO2, FAM92B, GRIN2C, IL-17REL, LILRA3, RAB40A and SCGB3A1) suggesting either; a) the antibodies are new and uncharacterized yet in WB/ICC; b) these proteins may not get expressed in SH-SY5Y cells; or c) may require bigger group size to see any significant knockdowns. (Raw blots are shown in **Supplementary Figure 11**). Data shown are GOI/Actin ratio or MFI mean ± SEM; n=3 biological replicates; *p<0.05; **p<0.01; ***p<0.005.

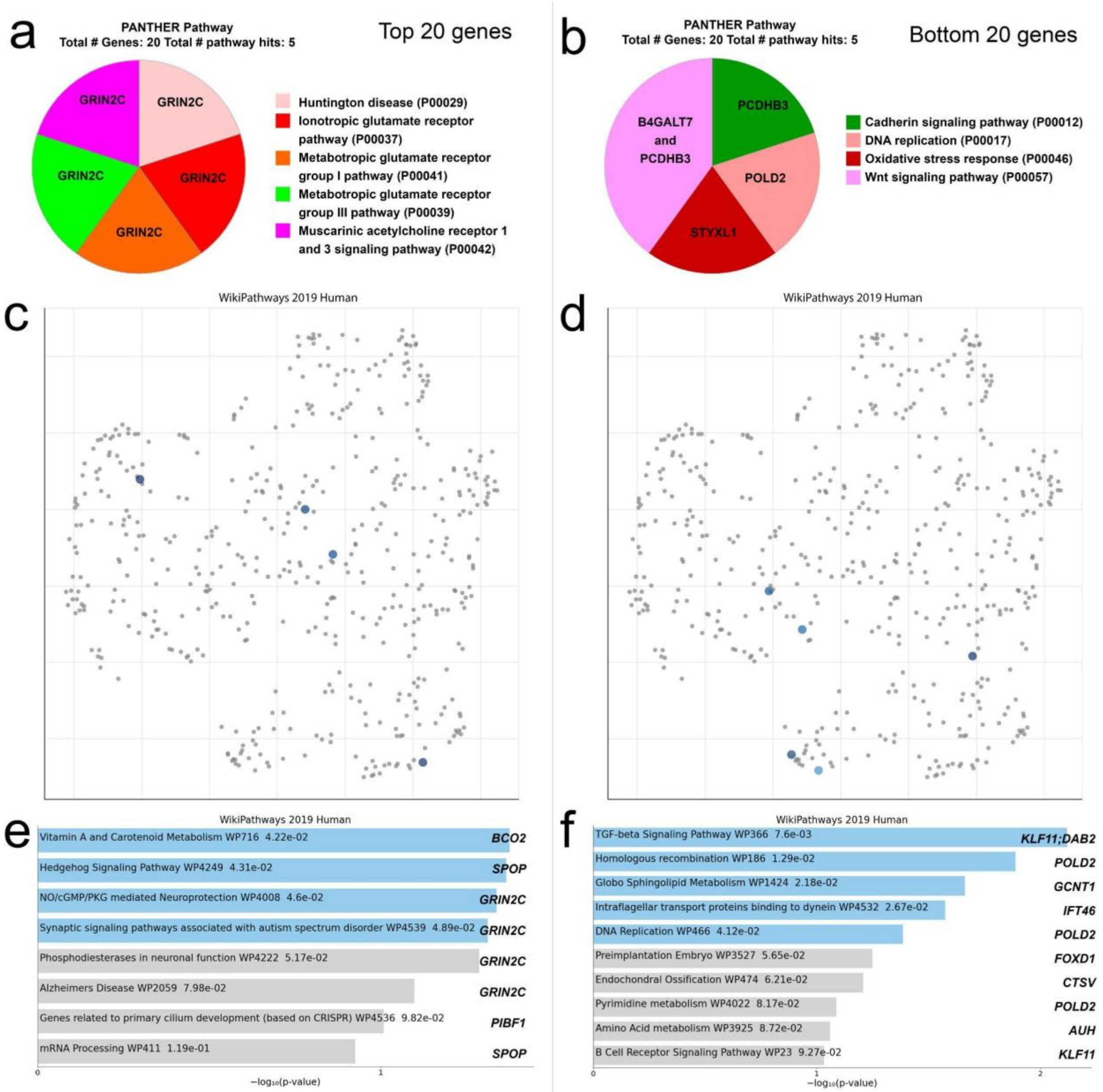

Supplementary Figure 5. Cross-reference for enrichment analysis data of the MPxgb(AD) prediction gene sets (Top-20 vs Bottom-20) a-b. Using gene function analysis with PANTHER[59] Classification System, GRIN2C is the only hit from the top 20 genes, representing neurological processes/function. B4GALT7, PCDHB3, STYXL1, and POLD2 were hits from the bottom 20 and classified in signaling pathways, oxidative stress response, and DNA replication. **c-d. Using EnrichR[60–62] Library - WikiPathways_2019_Human.** The scatterplot is organized so that similar gene sets are clustered together. The larger blue points represent significantly enriched terms - the darker the blue, the more significant the term and the smaller the p-value. The gray points are not significant. From top to bottom sets of genes, there does not seem to be any cluster overlap. **e-f.**The bar chart shows the top 10 enriched terms in the chosen library, along with their corresponding p-values. Colored bars correspond to terms with significant p-values (<0.05). Top 20 genes had four significant enriched terms, and four trending; Vitamin A and Carotenoid Metabolism (BCO2), Hedgehog Signaling Pathway (SPOP), NO/cGMP/PKG mediated Neuroprotection (GRIN2C), Synaptic signaling pathways associated with autism spectrum disorder (GRIN2C), Phosphodiesterases in neuronal function(GRIN2C), **Alzheimer's Disease (GRIN2C), Genes related to primary cilium development (PIBF1), and mRNA Processing (SPOP). The bottom genes had five significant enriched terms with five trending; TGF-beta Signaling Pathway (KLF11 and DAB2), Homologous recombination (POLD2), Globo Sphingolipid Metabolism (GCNT1), Intraflagellar transport proteins binding to dynein (IFT46), DNA Replication(POLD2).

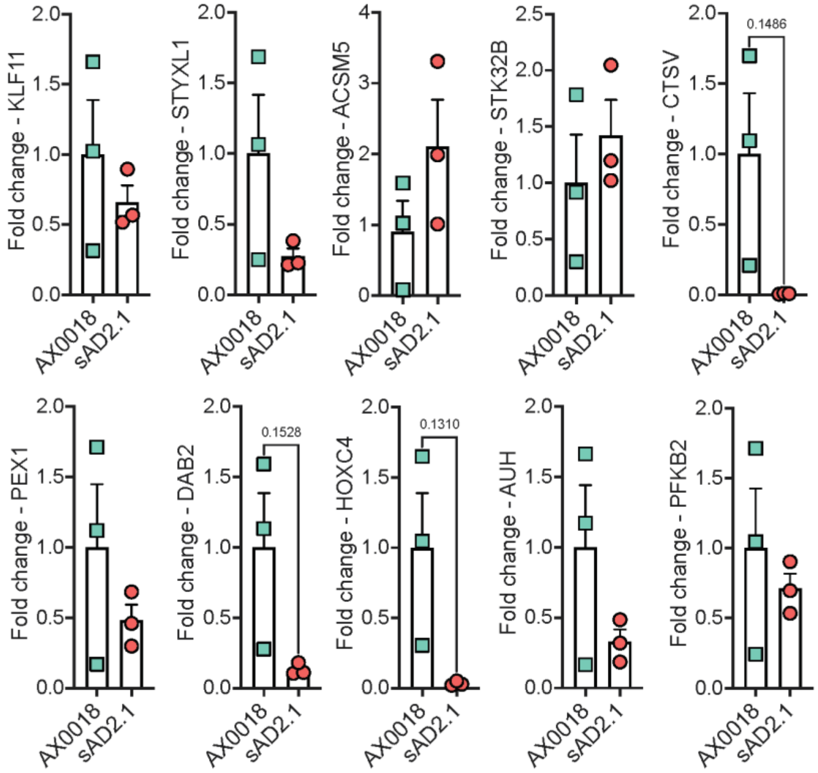

Supplementary Figure 6. mRNA levels of the least ten predicted GOI are not altered in human sAD2.1 iPSNs compared to control AX0018 iPSNs. qRT-PCR analysis showing no significant differences of mRNA levels in the least ten, in sAD2.1 iPSNs compared to AX0018 control iPSNs. Data shown are mean ± s.e.m; Two-tailed *t* tests welch-corrected; n=3 biological replicates; n=3 technical replicates.

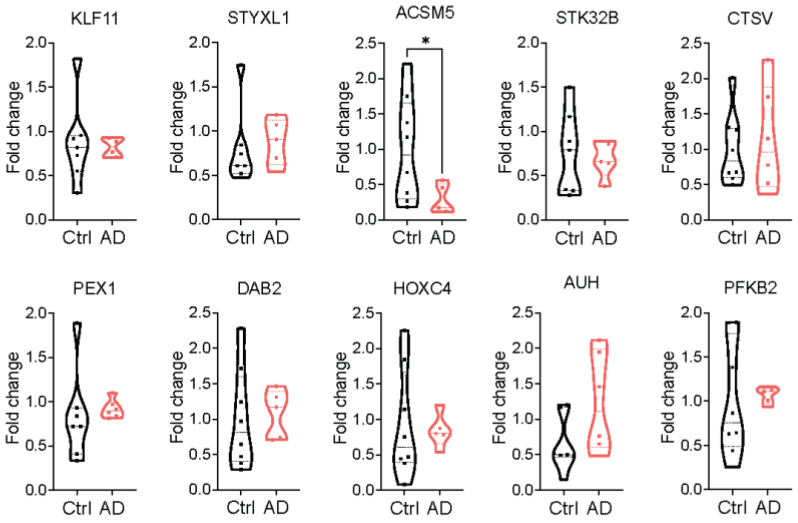

Supplementary Figure 7. Violin plots showing mRNA level of only ACSM5 is significantly altered in the post-mortem autopsy brains with Alzheimer's disease (AD). qRT-PCR analysis showing mRNA levels of ACSM5, but none of the other bottom GOI, are significantly altered in post-mortem temporal cortices of AD compared to age matched controls (Ctrl). Data shown are mean ± s.e.m; two-tailed *t* tests Welch-corrected and Tukey's for outlier removal; ACSM5's p value=0.0319; n=10 healthy controls and n=8 sporadic AD; n=3 technical replicates).

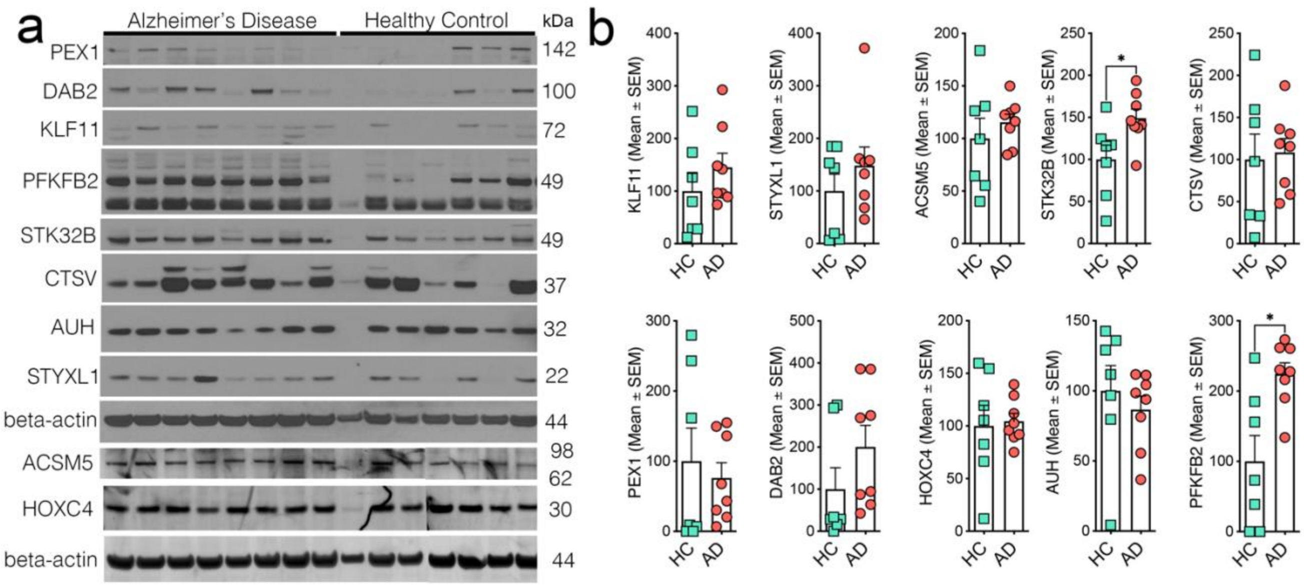

Supplementary Figure 8. Two proteins are significantly increased in post-mortem autopsy brains of human sporadic Alzheimer's disease. a-b. Western blot and quantifications showing significantly elevated levels of (P value of STK32B= 0.0346 and PFKFB2= 0.0140) in post-mortem temporal cortical samples of sporadic AD compared to age-matched healthy controls (HC). (Raw blots are shown in **Supplementary Figure 12**)**.** Data shown are mean ± s.e.m; two-tailed *t* tests Welch-corrected; *p<0.05; n=10 healthy controls and n=8 sporadic AD).

AT8 and Beta-Actin

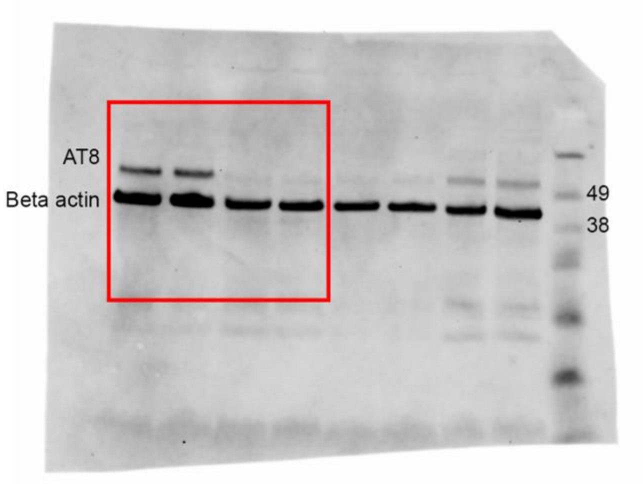

AT180

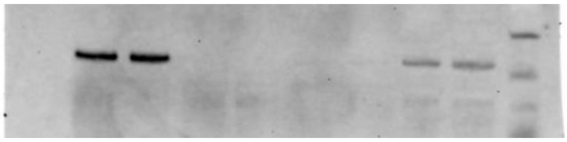

TAU12

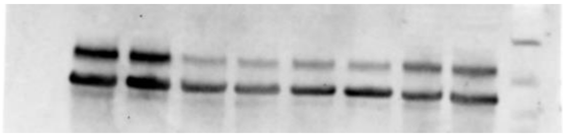

Supplementary Figure 9. Raw Blots for Figure 3c. Western blot showing significantly increased levels of total, pS199/pS202 (AT8), and pT231 (AT180) positive tau levels in sAD2.1 iPSNs compared to control AX0018 iPSNs.

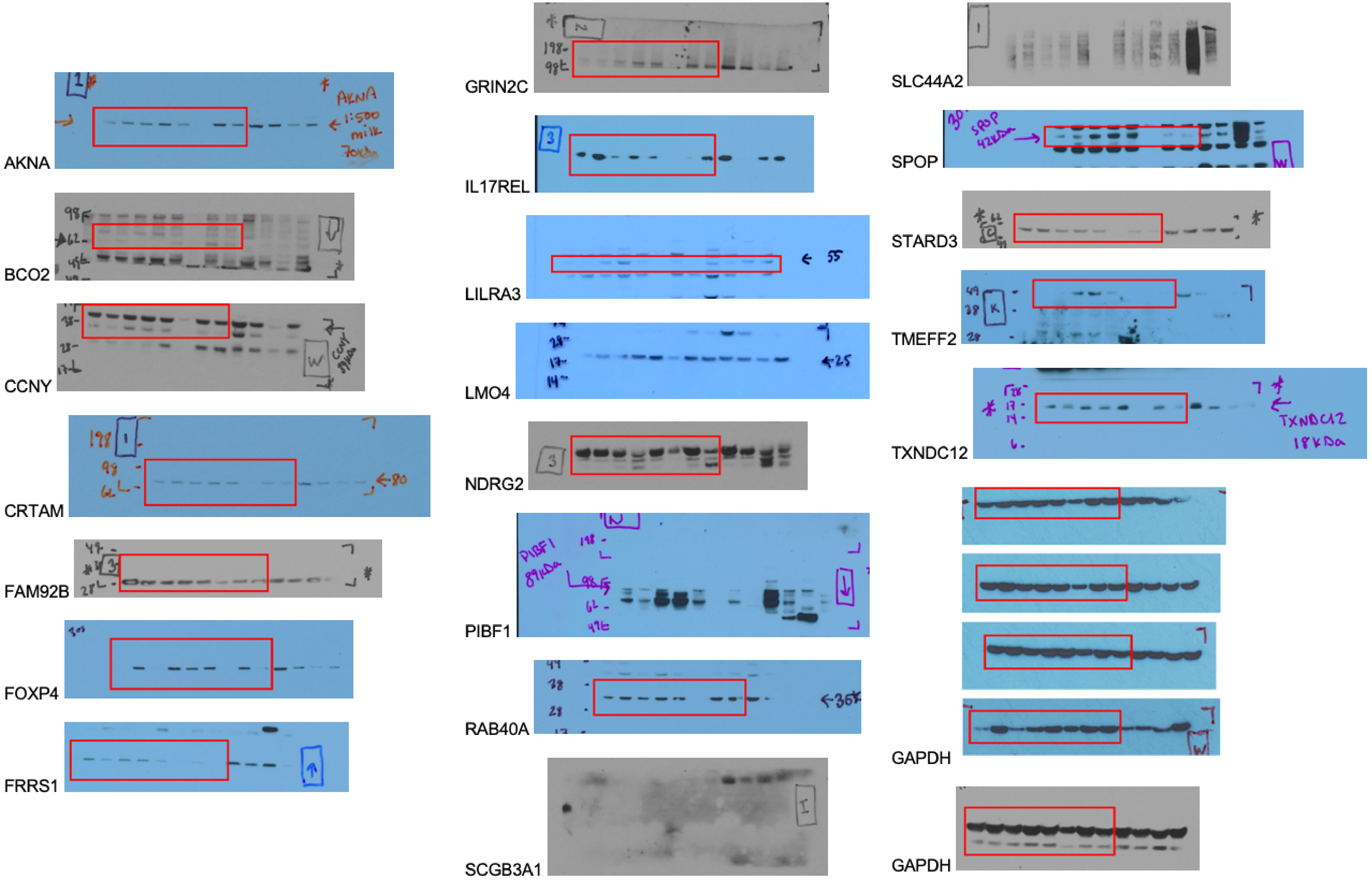

Supplementary Figure 10. Raw Blots for Figure 4a. Western showing significantly elevated levels of predicted proteins relevant to inflammatory pathways (PIBF1, CRTAM, FRRS1, and LILRA3), transcriptional regulation (FOXP4 and SPOP), metabolism (PIBF1 and STARD3), and others (TXNDC12 and FAM92B) in post-mortem temporal cortical samples of sporadic AD compared to age-matched healthy controls (HC). Note that SCGB3A1 and SLC44A2 were not detectable.

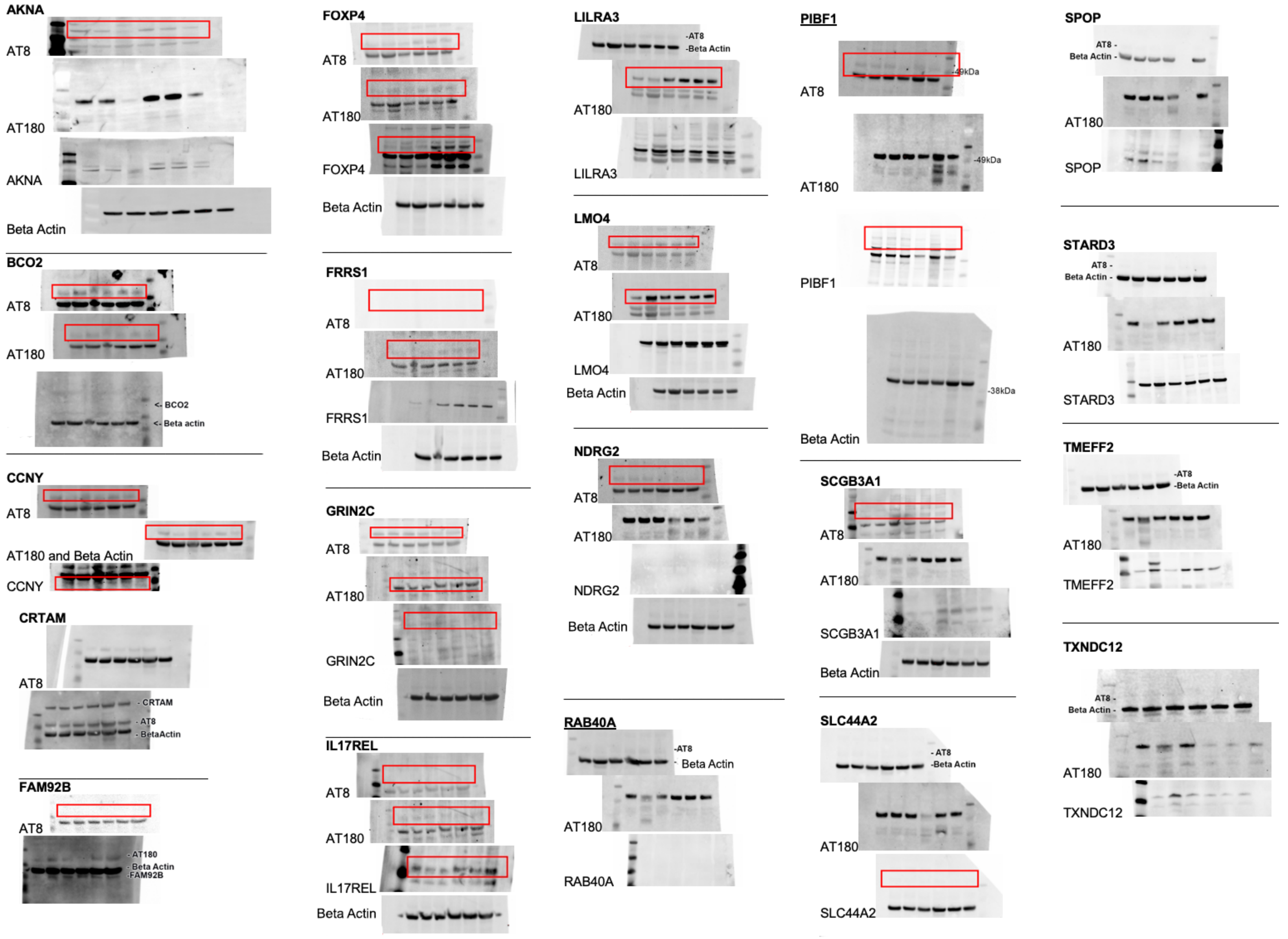

Supplementary Figure 11. Raw Blots for Figure 5 and Supplementary Figure 4. Western blot was performed for knockdown confirmation, and changes in AT8 and AT180 levels with Beta-Actin as loading control.

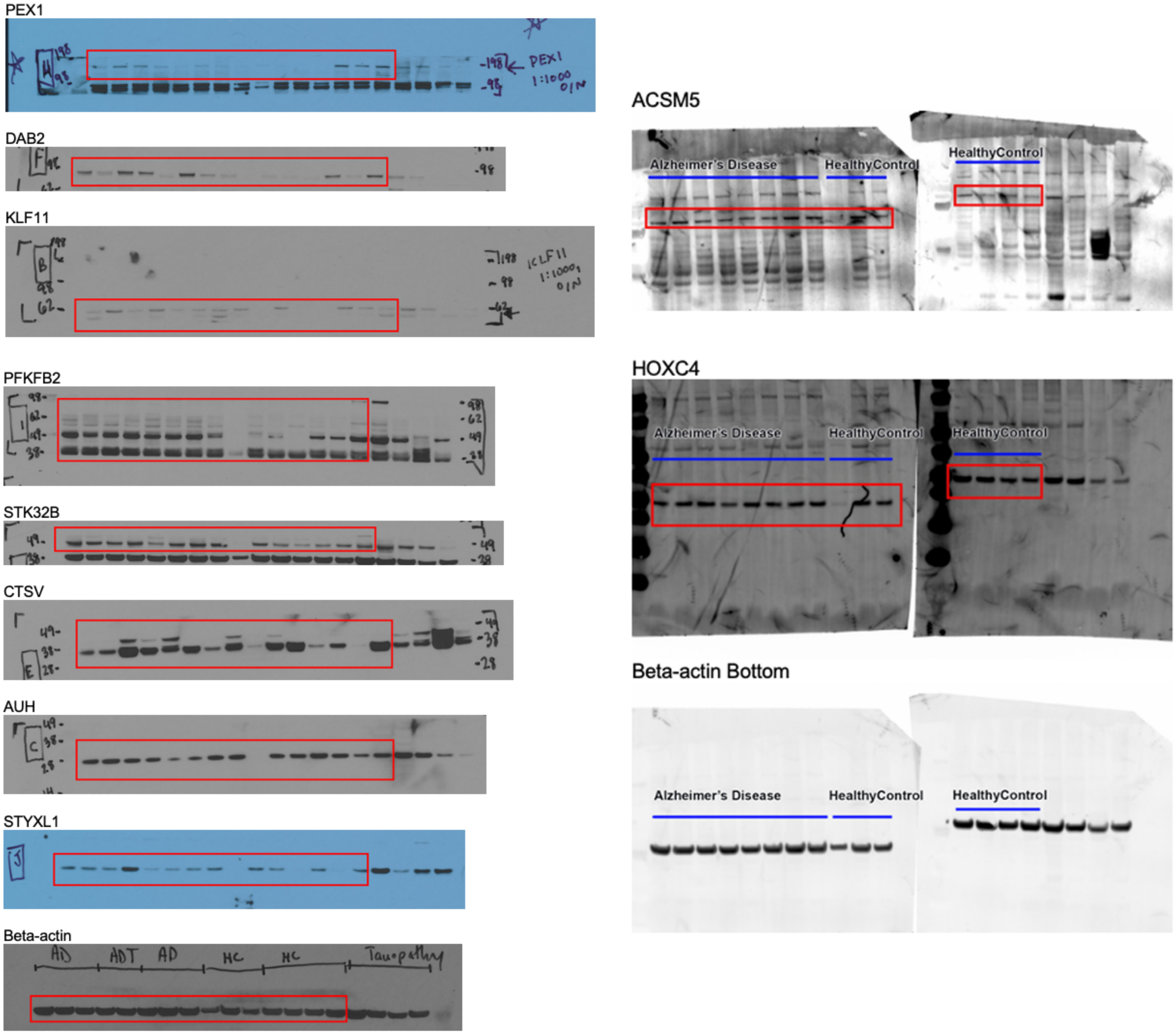

Supplementary Figure 12. Raw Blots for Supplementary Figure 8a. Western blot showing significantly elevated levels of (P value of STK32B= 0.0346 and PFKFB2= 0.0140) in post-mortem temporal cortical samples of sporadic AD compared to age-matched healthy controls (HC)

SUPPLEMENTARY NOTES

Supplementary Note 1: ML AD genes with their previously reported functional annotations

1. **AKNA** – AKNA is a transcription factor that binds to A/T-rich promoters, more specifically activates the expression of the CD40 receptor and its ligand CD40L1. Typically, AKNA has been critically associated for antigen-dependent-B-cell development. However, it has been shown that (CD40-CD40L) interaction supports pathogenesis of AD2.
2. **FRSS1** – FRSS1 is a Ferric-chelate reductase that reduces Fe^{3+} to Fe^{2+} before its transport from the endosome to the cytoplasm[3]. Interestingly, age-related dysregulation of brain iron homeostasis leads to abnormal iron accumulation FRRS1L[4] also plays an important role in Glutamatergic synaptic transmission[5]. Another study suggests under proinflammatory responses, it creates a greater uptake of iron in brain microglia[6].
3. **RAB40A** – RAB40A encodes a member of the Rab40 subfamily of Rab small GTP-binding proteins that contain a C-terminal suppressor of cytokine signaling box[7]. Many studies have shown the roles of Rab GTPase dysregulation in AD pathogenesis[8].
4. **STARD3** – STARD3, StAR Related Lipid Transfer Domain Containing 3, is a sterol-binding protein that mediates cholesterol transport from the endoplasmic reticulum (ER) to endosomes[9–12]. Cholesterol transport and metabolism has been highly implicated in AD pathogenesis[13,14].
5. **PIBF1** – PIBF1, Progesterone Immunomodulatory Binding Factor 1, encodes a protein that is induced by the steroid hormone progesterone. PIBF1 has been shown in multiple aspects of the immune system to promote normal pregnancy including cytokine synthesis, natural killer (NK) cell activity, and arachidonic acid metabolism[15]. There have been studies that suggest depletion of estrogens and progestogens increase susceptibility to AD pathogenesis[16]. On the other hand, progestogens provide a protective phenotype in microglia cells[17].
6. **TXNDC12** – TXNDC12, Thioredoxin Domain Containing 12, mediates disulfide bond formation in the ER and plays an important role in cellular defense against prolonged ER stress[18]. Some studies have suggested that levels of Thioredoxin are significantly increased in the early stages of AD[19]. Yet others suggest the opposite[20]. Another study characterized the association with NLRP3 inflammasome activation via Thioredoxin-Interacting Protein (TXNIP)[21].
7. **SCGB3A1** – SCGB3A1, Secretoglobin Family 3A Member 1, Secreted cytokine-like protein. Alteration of secretoglobins gene expression, may contribute to immunoregulatory perturbations commonly seen in chronic airway disease[22].
8. **NDRG2** – NDRG2, N-Myc Downstream-Regulated Gene 2 Protein which belongs to the alpha/beta hydrolase superfamily. This gene may play a role in glioblastoma carcinogenesis[23]. It also has been shown to be involved in dendritic cell and neuron differentiation[24,25]. One study implicated that Ndrg2 may regulate astroglial activation[26]. Finally, at the clinical level, it has been associated with the pathogenesis of AD[27].
9. **CCNY** – CCNY, Cyclin Y, controls cell division cycle and regulates cyclin-dependent kinases[28]. It is also a positive regulatory subunit of the cyclin-dependent kinases CDK14/PFTK1 and CDK16[29]. Acts as a positive cell-cycle regulator of Wnt signaling pathway during the G2/M phase by recruiting CDK14/PFTK1 to the plasma membrane and promoting phosphorylation of LRP6. However, Wnt signaling loss has been shown to drive AD pathogenesis[30].
10. **FAM92B** – FAM92B, Family with Sequence Similarity 92 Member B, may play a role in ciliogenesis via cell projection organization[31].
11. **GRIN2C** – GRIN2C, Glutamate Ionotropic Receptor NMDA Type Subunit 2C, encodes a subunit of the N-methyl-D-aspartate (NMDA) receptor, which is a subtype of ionotropic glutamate receptor[32]. NMDA receptors are important for learning, memory, and synaptic development[33]. Alterations in the subunit composition of the receptor are associated with pathophysiological conditions such as Parkinson's disease, Alzheimer's disease, depression, and schizophrenia[34,35].
12. **FOXP4** – FOXP4, Forkhead Box P4, belongs to subfamily P of the forkhead box (FOX) transcription factor family. Forkhead box transcription factors play important roles in AD, mostly in the subfamily O[36]. However, many members of the forkhead box gene family, including members of subfamily P, have roles in mammalian oncogenesis[37].
13. **SPOP** – SPOP, Speckle Type BTB/POZ Protein, encodes a protein that has been shown to repress the death-associated protein 6 (DAXX) at the transcriptional level, which interacts with histone deacetylase, core

histones, and other histone-associated proteins (PMID: 18997279). Mostly, SPOP has been associated with cancer[38]. But has not yet been implicated with AD.

14. **IL17RE**L – IL17REL, Interleukin 17 Receptor E Like, is a functional receptor for IL17C. IL17REL is a homolog of IL17RE, associated with the pathway to initiate the Th2-mediated immune response[39,40].
15. **CRTAM** – CRTAM, Cytotoxic and Regulatory T Cell Molecule, encodes a type I transmembrane protein with V and C1-like Ig domains[41]. The CRTAM gene is upregulated in CD4+ and CD8+ T cells[42].
16. **TMEFF2** – TMEFF2, Transmembrane Protein with EGF Like And Two Follistatin Like Domains 2, encodes a member of the tomoregulin family of transmembrane proteins. This protein has been debated between an oncogene or tumor suppressor depending on the cellular context in cancer[43]. But it has also been shown to be protective in AD via binding of Amyloid-B oligomers[44]. In addition, this study suggests an endurance element for hippocampal and mesencephalic neurons[45].
17. **BCO2** – BCO2, Beta-Carotene Oxygenase 2, encodes an enzyme which oxidizes carotenoids such as beta-carotene during the biosynthesis of vitamin A. Some studies have shown that intake of certain vitamins serves as a protective attribute to AD[46,47]. However, one study shows that increased levels of plasma beta carotene strongly correlated with risk in AD[48].
18. **LMO4** – LMO4, LIM Domain Only 4, is a transcription regulator encoded with a cysteine-rich protein that contains two LIM domains but lacks a DNA-binding homeodomain[49]. One group distinguished lower levels of LMO4 in AD brains directly correlated with the amount of NFTs present[50].
19. **LILRA3** – LILRA3, Leukocyte Immunoglobulin Like Receptor A3, encodes a member of a family of immunoreceptors that are primarily expressed in monocytes and B cells, and at lower levels in dendritic cells and natural killer cells[51,52]. LILRA3 binds with high affinity to the surface of monocytes, leading to abolish LPS-induced TNF-alpha production by monocytes[53].
20. **SLC44A2** – SLC44A2, Solute Carrier Family 44 Member 2, is a carrier protein. Also known as CTL2, Choline transporter-like protein 2. Depending on the isoform and tissue expression determines the functions[54]. One study has also associated it to cholinergic neurons[55].

---

21. **ACSM5** – ACSM5, Acyl-CoA Synthetase Medium Chain Family Member 5, is related to Cytochrome P450 and fatty acid beta-oxidation (peroxisome) pathways[56] and includes ATP and GTP binding, fatty acid ligase, acyl-CoA ligase and butyrate-CoA ligase activity[57]. Herpetic Whitlow[58] is known to be associated with ACSM5.
22. **STK32B** – STK32B, Serine/Threonine Kinase 32B, has been associated with Ellis-van Creveld syndrome, an autosomal recessive skeletal dysplasia. STK32B is related to pathways in sweet taste signaling, transferase activity, transferring phosphorus-containing groups and protein tyrosine kinase activity.
23. **PFKFB2** – PFKFB2, 6-Phosphofructo-2-Kinase/Fructose-2,6-Bisphosphatase 2, is involved in both the synthesis and degradation of fructose-2,6-bisphosphate, a regulatory molecule that controls glycolysis in eukaryotes.[57]

Supplementary References

1. Siddiqa, A. *et al.* Regulation of CD40 and CD40 ligand by the AT-hook transcription factor AKNA. *Nature* **410**, 383–387 (2001).

2. Tan, J., Town, T. & Mullan, M. CD40-CD40L interaction in Alzheimer’s disease. *Curr. Opin. Pharmacol.* **2**, 445–451 (2002).

3. Vargas, J. D. *et al.* Stromal cell-derived receptor 2 and cytochrome b561 are functional ferric reductases. *Biochim. Biophys. Acta* **1651**, 116–123 (2003).

4. Oshiro, S., Morioka, M. S. & Kikuchi, M. Dysregulation of iron metabolism in Alzheimer’s disease, Parkinson's disease, and amyotrophic lateral sclerosis. *Adv. Pharmacol. Sci.* **2011**, 378278 (2011).

5. Han, W., Wang, H., Li, J., Zhang, S. & Lu, W. Ferric Chelate Reductase 1 Like Protein (FRRS1L) Associates with Dynein Vesicles and Regulates Glutamatergic Synaptic Transmission. *Front. Mol. Neurosci.* **10**, 402 (2017).
6. McCarthy, R. C. *et al.* Inflammation-induced iron transport and metabolism by brain microglia. *J. Biol. Chem.* **293**, 7853–7863 (2018).
7. Pereira-Leal, J. B. & Seabra, M. C. Evolution of the Rab family of small GTP-binding proteins. *J. Mol. Biol.* **313**, 889–901 (2001).
8. Zhang, X., Huang, T. Y., Yancey, J., Luo, H. & Zhang, Y.-W. Role of Rab GTPases in Alzheimer's Disease. *ACS Chem. Neurosci.* **10**, 828–838 (2019).
9. Alpy, F. *et al.* The steroidogenic acute regulatory protein homolog MLN64, a late endosomal cholesterol-binding protein. *J. Biol. Chem.* **276**, 4261–4269 (2001).
10. van der Kant, R., Zondervan, I., Janssen, L. & Neefjes, J. Cholesterol-binding molecules MLN64 and ORP1L mark distinct late endosomes with transporters ABCA3 and NPC1. *J. Lipid Res.* **54**, 2153–2165 (2013).
11. Liapis, A., Chen, F. W., Davies, J. P., Wang, R. & Ioannou, Y. A. MLN64 transport to the late endosome is regulated by binding to 14-3-3 via a non-canonical binding site. *PLoS One* **7**, e34424 (2012).
12. Wilhelm, L. P. *et al.* STARD3 mediates endoplasmic reticulum-to-endosome cholesterol transport at membrane contact sites. *EMBO J.* **36**, 1412–1433 (2017).
13. Martins, I. J. *et al.* Cholesterol metabolism and transport in the pathogenesis of Alzheimer's disease. *J. Neurochem.* **111**, 1275–1308 (2009).
14. Chang, T.-Y., Yamauchi, Y., Hasan, M. T. & Chang, C. Cellular cholesterol homeostasis and Alzheimer's disease. *J. Lipid Res.* **58**, 2239–2254 (2017).
15. Hudić, I. *et al.* Lower Urinary and Serum Progesterone-Induced Blocking Factor in Women with Preterm Birth. *J. Reprod. Immunol.* **117**, 66–69 (2016).
16. Pike, C. J., Carroll, J. C., Rosario, E. R. & Barron, A. M. Protective actions of sex steroid hormones in Alzheimer's disease. *Front. Neuroendocrinol.* **30**, 239–258 (2009).
17. Lei, B. *et al.* Anti-inflammatory effects of progesterone in lipopolysaccharide-stimulated BV-2

microglia. *PLoS One* **9**, e103969 (2014).

18. Jeong, W., Lee, D.-Y., Park, S. & Rhee, S. G. ERp16, an endoplasmic reticulum-resident thiol-disulfide oxidoreductase: biochemical properties and role in apoptosis induced by endoplasmic reticulum stress. *J. Biol. Chem.* **283**, 25557–25566 (2008).
19. Arodin, L. *et al.* Alteration of thioredoxin and glutaredoxin in the progression of Alzheimer's disease. *J. Alzheimers. Dis.* **39**, 787–797 (2014).
20. Lovell, M. A., Xie, C., Gabbita, S. P. & Markesbery, W. R. Decreased thioredoxin and increased thioredoxin reductase levels in Alzheimer's disease brain. *Free Radic. Biol. Med.* **28**, 418–427 (2000).
21. Li, L. *et al.* Thioredoxin-Interacting Protein (TXNIP) Associated NLRP3 Inflammasome Activation in Human Alzheimer's Disease Brain. *J. Alzheimers. Dis.* **68**, 255–265 (2019).
22. Reynolds, S. D., Reynolds, P. R., Pryhuber, G. S., Finder, J. D. & Stripp, B. R. Secretoglobins SCGB3A1 and SCGB3A2 define secretory cell subsets in mouse and human airways. *Am. J. Respir. Crit. Care Med.* **166**, 1498–1509 (2002).
23. Deng, Y. *et al.* N-Myc downstream-regulated gene 2 (NDRG2) inhibits glioblastoma cell proliferation. *Int. J. Cancer* **106**, 342–347 (2003).
24. Zhou, R. H. *et al.* Characterization of the human NDRG gene family: a newly identified member, NDRG4, is specifically expressed in brain and heart. *Genomics* **73**, 86–97 (2001).
25. Okuda, T. & Kondoh, H. Identification of new genes ndr2 and ndr3 which are related to Ndr1/RTP/Drg1 but show distinct tissue specificity and response to N-myc. *Biochem. Biophys. Res. Commun.* **266**, 208–215 (1999).
26. Takeichi, T. *et al.* The effect of Ndrg2 expression on astroglial activation. *Neurochem. Int.* **59**, 21–27 (2011).
27. Mitchelmore, C. *et al.* NDRG2: a novel Alzheimer's disease associated protein. *Neurobiol. Dis.* **16**, 48–58 (2004).
28. Li, X., Wang, X., Liu, G., Li, R. & Yu, L. Identification and characterization of cyclin X which activates transcriptional activities of c-Myc. *Mol. Biol. Rep.* **36**, 97–103 (2009).
29. Jiang, M., Gao, Y., Yang, T., Zhu, X. & Chen, J. Cyclin Y, a novel membrane-associated cyclin,

interacts with PFTK1. *FEBS Lett.* **583**, 2171–2178 (2009).

30. Tapia-Rojas, C. & Inestrosa, N. C. Loss of canonical Wnt signaling is involved in the pathogenesis of Alzheimer's disease. *Neural Regeneration Res.* **13**, 1705–1710 (2018).
31. Li, F.-Q. *et al.* BAR Domain-Containing FAM92 Proteins Interact with Chibby1 To Facilitate Ciliogenesis. *Mol. Cell. Biol.* **36**, 2668–2680 (2016).
32. Hackos, D. H. *et al.* Positive Allosteric Modulators of GluN2A-Containing NMDARs with Distinct Modes of Action and Impacts on Circuit Function. *Neuron* **89**, 983–999 (2016).
33. Morris, R. G. M. NMDA receptors and memory encoding. *Neuropharmacology* **74**, 32–40 (2013).
34. Wang, R. & Reddy, P. H. Role of Glutamate and NMDA Receptors in Alzheimer's Disease. *J. Alzheimers. Dis.* **57**, 1041–1048 (2017).
35. Zhang, Y., Li, P., Feng, J. & Wu, M. Dysfunction of NMDA receptors in Alzheimer's disease. *Neurol. Sci.* **37**, 1039–1047 (2016).
36. Manolopoulos, K. N., Klotz, L.-O., Korsten, P., Bornstein, S. R. & Barthel, A. Linking Alzheimer's disease to insulin resistance: the FoxO response to oxidative stress. *Mol. Psychiatry* **15**, 1046–1052 (2010).
37. Sjöblom, T. *et al.* The consensus coding sequences of human breast and colorectal cancers. *Science* **314**, 268–274 (2006).
38. Blattner, M. *et al.* SPOP Mutation Drives Prostate Tumorigenesis In Vivo through Coordinate Regulation of PI3K/mTOR and AR Signaling. *Cancer Cell* **31**, 436–451 (2017).
39. Wu, B., Jin, M., Zhang, Y., Wei, T. & Bai, Z. Evolution of the IL17 receptor family in chordates: a new subfamily IL17REL. *Immunogenetics* **63**, 835–845 (2011).
40. Gaffen, S. L. Structure and signalling in the IL-17 receptor family. *Nat. Rev. Immunol.* **9**, 556–567 (2009).
41. Yeh, J.-H., Sidhu, S. S. & Chan, A. C. Regulation of a late phase of T cell polarity and effector functions by Crtam. *Cell* **132**, 846–859 (2008).
42. Boles, K. S., Barchet, W., Diacovo, T., Cella, M. & Colonna, M. The tumor suppressor TSLC1/NECL-2 triggers NK-cell and CD8+ T-cell responses through the cell-surface receptor CRTAM. *Blood* **106**, 779–786 (2005).

43. Ali, N. & Knaüper, V. Phorbol ester-induced shedding of the prostate cancer marker transmembrane protein with epidermal growth factor and two follistatin motifs 2 is mediated by the disintegrin and metalloproteinase-17. *J. Biol. Chem.* **282**, 37378–37388 (2007).

44. Hong, H.-S. *et al.* Tomoregulin (TMEFF2) Binds Alzheimer's Disease Amyloid-β (Aβ) Oligomer and AβPP and Protects Neurons from Aβ-Induced Toxicity. *J. Alzheimers. Dis.* **48**, 731–743 (2015).

45. Horie, M. *et al.* Identification and characterization of TMEFF2, a novel survival factor for hippocampal and mesencephalic neurons. *Genomics* **67**, 146–152 (2000).

46. Obulesu, M., Dowlathabad, M. R. & Bramhachari, P. V. Carotenoids and Alzheimer's disease: an insight into therapeutic role of retinoids in animal models. *Neurochem. Int.* **59**, 535–541 (2011).

47. Li, F.-J., Shen, L. & Ji, H.-F. Dietary intakes of vitamin E, vitamin C, and β-carotene and risk of Alzheimer's disease: a meta-analysis. *J. Alzheimers. Dis.* **31**, 253–258 (2012).

48. Stuerenburg, H. J., Ganzer, S. & Müller-Thomsen, T. Plasma beta carotene in Alzheimer's disease. Association with cerebrospinal fluid beta-amyloid 1-40, (Abeta40), beta-amyloid 1-42 (Abeta42) and total Tau. *Neuro Endocrinol. Lett.* **26**, 696–698 (2005).

49. Holik, A. Z. *et al.* The LIM-domain only protein 4 contributes to lung epithelial cell proliferation but is not essential for tumor progression. *Respir. Res.* **16**, 67 (2015).

50. Leuba, G. *et al.* Differential expression of LMO4 protein in Alzheimer's disease. *Neuropathol. Appl. Neurobiol.* **30**, 57–69 (2004).

51. Lee, T. H. Y. *et al.* Glycosylation in a mammalian expression system is critical for the production of functionally active leukocyte immunoglobulin-like receptor A3 protein. *J. Biol. Chem.* **288**, 32873–32885 (2013).

52. Ryu, M. *et al.* LILRA3 binds both classical and non-classical HLA class I molecules but with reduced affinities compared to LILRB1/LILRB2: structural evidence. *PLoS One* **6**, e19245 (2011).

53. Low, H. Z. *et al.* TLR8 regulation of LILRA3 in monocytes is abrogated in human immunodeficiency virus infection and correlates to CD4 counts and virus loads. *Retrovirology* **13**, 15 (2016).

54. Kommareddi, P. K. *et al.* Isoforms, expression, glycosylation, and tissue distribution of

CTL2/SLC44A2. *Protein J.* **29**, 417–426 (2010).

55. O'Regan, S. *et al.* An electric lobe suppressor for a yeast choline transport mutation belongs to a new family of transporter-like proteins. *Proc. Natl. Acad. Sci. U. S. A.* **97**, 1835–1840 (2000).
56. Belinky, F. *et al.* PathCards: multi-source consolidation of human biological pathways. *Database* **2015**, (2015).
57. Sheils, T. K. *et al.* TCRD and Pharos 2021: mining the human proteome for disease biology. *Nucleic Acids Res.* **49**, D1334–D1346 (2021).
58. Rappaport, N. *et al.* MalaCards: an amalgamated human disease compendium with diverse clinical and genetic annotation and structured search. *Nucleic Acids Res.* **45**, D877–D887 (2017).
59. Mi, H. *et al.* Protocol Update for large-scale genome and gene function analysis with the PANTHER classification system (v.14.0). *Nat. Protoc.* **14**, 703–721 (2019).
60. Xie, Z. *et al.* Gene set knowledge discovery with Enrichr. *Curr Protoc* **1**, e90 (2021).
61. Kuleshov, M. V. *et al.* Enrichr: a comprehensive gene set enrichment analysis web server 2016 update. *Nucleic Acids Res.* **44**, W90–W97 (2016).
62. Chen, E. Y. *et al.* Enrichr: interactive and collaborative HTML5 gene list enrichment analysis tool. *BMC Bioinformatics* **14**, 128 (2013).
63. Clarke, D. J. B. *et al.* Appyters: Turning Jupyter Notebooks into data-driven web apps. *Patterns (N Y)* **2**, 100213 (2021).
