# Supplementary material for: Machine learning prediction and tau-based screening identifies potential Alzheimer’s disease genes relevant to immunity
Source: Commun Biol. 2022 Feb 11;5:125. doi: 10.1038/s42003-022-03068-7 (PMC8837797; doi:10.1038/s42003-022-03068-7)
Supplement: Supplementary file 3 — Reporting Summary [file 42003_2022_3068_MOESM3_ESM.pdf]

nature portfolio

Corresponding author(s): Tudor I. Oprea

Last updated by author(s): Jan 11, 2022

Reporting Summary

Nature Portfolio wishes to improve the reproducibility of the work that we publish. This form provides structure for consistency and transparency in reporting. For further information on Nature Portfolio policies, see our Editorial Policies and the Editorial Policy Checklist.

Statistics

For all statistical analyses, confirm that the following items are present in the figure legend, table legend, main text, or Methods section.

n/a	Confirmed	
☐	☒	The exact sample size (n) for each experimental group/condition, given as a discrete number and unit of measurement
☐	☒	A statement on whether measurements were taken from distinct samples or whether the same sample was measured repeatedly
☐	☒	The statistical test(s) used AND whether they are one- or two-sided<br>*Only common tests should be described solely by name; describe more complex techniques in the Methods section.*
☒	☐	A description of all covariates tested
☒	☐	A description of any assumptions or corrections, such as tests of normality and adjustment for multiple comparisons
☐	☒	A full description of the statistical parameters including central tendency (e.g. means) or other basic estimates (e.g. regression coefficient) AND variation (e.g. standard deviation) or associated estimates of uncertainty (e.g. confidence intervals)
☐	☒	For null hypothesis testing, the test statistic (e.g. F, t, r) with confidence intervals, effect sizes, degrees of freedom and P value noted<br>*Give P values as exact values whenever suitable.*
☒	☐	For Bayesian analysis, information on the choice of priors and Markov chain Monte Carlo settings
☒	☐	For hierarchical and complex designs, identification of the appropriate level for tests and full reporting of outcomes
☒	☐	Estimates of effect sizes (e.g. Cohen's d, Pearson's r), indicating how they were calculated

Our web collection on statistics for biologists contains articles on many of the points above.

Software and code

Policy information about availability of computer code

Data collection	code: https://github.com/unmtransinfo/ProteinGraphML<br><br>For experimental: AlphaEase and BioRad Gel Doc system were used to collect analyzed Western Blot Data. StepOnePlus qRT-PCR for mRNA expression levels.
Data analysis	GraphPad Prism (version 8.0), SAS v9.4 and Statemate were used for statistical analyses and power calculations, respectively.

For manuscripts utilizing custom algorithms or software that are central to the research but not yet described in published literature, software must be made available to editors and reviewers. We strongly encourage code deposition in a community repository (e.g. GitHub). See the Nature Portfolio guidelines for submitting code & software for further information.

Data

Policy information about availability of data

All manuscripts must include a data availability statement. This statement should provide the following information, where applicable:

- Accession codes, unique identifiers, or web links for publicly available datasets
- A description of any restrictions on data availability
- For clinical datasets or third party data, please ensure that the statement adheres to our policy

For database/code: http://juniper.health.unm.edu/tcrd/ and https://github.com/unmtransinfo/ProteinGraphML

Raw experimental data is stored in several different places; 1.) https://figshare.com/projects/Machine_learning_prediction_and_tau-based_screening_identifies_potential_Alzheimer_s_disease_genes_relevant_to_immunity/127145

2.) google docs
and 3.) University of New Mexico Health Sciences Center cloud storage drive. It is also available upon request by contacting the corresponding authors.

Field-specific reporting

Please select the one below that is the best fit for your research. If you are not sure, read the appropriate sections before making your selection.

☒ Life sciences ☐ Behavioural & social sciences ☐ Ecological, evolutionary & environmental sciences

For a reference copy of the document with all sections, see nature.com/documents/nr-reporting-summary-flat.pdf

Life sciences study design

All studies must disclose on these points even when the disclosure is negative.

Sample size	The coefficient of variance for our studies on hyper-phosphorylated tau is ~19% and power analysis (Statmate) suggest that a group size of 3 is sufficient to detect 33% mean ratio (pTau - AT8 or AT180/GAPDH) in two experimental groups in biochemical analysis.
Data exclusions	Any data that was excluded was only excluded if the data point fell outside two standard deviations outside of the mean of the data set, and these criterion were per-determined prior to experiments and the exclusions were calculated in Prism 8.0 with 'Identify Outlier' function. As well as confirmed on excel function.
Replication	Data generated for this study was replicated across three or more biological replicates and three technical replicates.
Randomization	Samples (autopsy samples and cell culture samples) were randomized to different groups prior to any treatment/experiment/analyses.
Blinding	Experimenters remained blinded to treatments until the end of the experiment.

Reporting for specific materials, systems and methods

We require information from authors about some types of materials, experimental systems and methods used in many studies. Here, indicate whether each material, system or method listed is relevant to your study. If you are not sure if a list item applies to your research, read the appropriate section before selecting a response.

Materials & experimental systems

n/a	Involved in the study	
☐	☒	Antibodies
☐	☒	Eukaryotic cell lines
☒	☐	Palaeontology and archaeology
☒	☐	Animals and other organisms
☒	☐	Human research participants
☒	☐	Clinical data
☒	☐	Dual use research of concern

Methods

n/a	Involved in the study	
☒	☐	ChIP-seq
☒	☐	Flow cytometry
☒	☐	MRI-based neuroimaging

Antibodies

Antibodies used

Antibody Species Company and Catalog # Dilutions
AKNA Rabbit Abcam#ab220392 1:1000 (WB)
1:500 (IF)
BCO2 Rabbit Thermofisher#PA5-24527 1:1000 (WB)
1:250 (IF)
CCNY Rabbit Thermofisher#PA5-23644 1:1000 (WB)
1:250 (IF)
CRTAM Mouse SantaCruz# sc-390581 1:1000 (WB)
1:500 (IF)
FAM92B Rabbit Thermofisher#PA5-59398 1:1000 (WB)
1:250 (IF)
FOXP4 Rabbit Thermofisher#PA5-49682 1:1000 (WB)
1:250 (IF)
FRRS1 Rabbit Abcam# ab121538 1:1000 (WB)
1:500 (IF)
GRIN2C Rabbit Thermofisher#OPA1-04020 1:1000 (WB)
1:250 (IF)
IL17REL Rabbit Abcam# ab126399 1:1000 (WB)
1:500 (IF)
LILRA3 Rabbit Thermofisher# PA5-28902 1:1000 (WB)

	1:250 (IF)<br>LMO4 Rabbit Thermofisher# PA5-24248 1:1000 (WB)<br>1:250 (IF)<br>NDRG2 Mouse SantaCruz# sc-376202 1:1000 (WB)<br>1:500 (IF)<br>PIBF1 Rabbit Thermofisher# PA5-34514 1:1000 (WB)<br>1:500 (IF)<br>RAB40A Rabbit Thermofisher# PA5-69848 1:1000 (WB)<br>1:250 (IF)<br>SCGB3A1 Mouse Abcam# ab201604 1:1000 (WB)<br>1:500 (IF)<br>SLC44A2 Rabbit Thermofisher# PA5-67127 1:1000 (WB)<br>1:500 (IF)<br>SPOP Rabbit Thermofisher# PA5-28522 1:1000 (WB)<br>1:250 (IF)<br>STARD3 Rabbit Thermofisher#PA1-562 1:1000 (WB)<br>1:250 (IF)<br>TMEFF2 Rabbit Thermofisher#PA5-53327 1:1000 (WB)<br>1:250 (IF)<br>TXNDC12 Rabbit Thermofisher#PA5-24798 1:1000 (WB)<br>1:250 (IF)<br>Beta-actin Mouse Abcam#ab8226 1:10,000 (WB)<br>AT180 Mouse Thermo Scientific, MN1040 1:5000 (WB)<br>AT8 Mouse Thermo Scientific, MN1020 1:10,000 (WB)<br>GAPDH Mouse Millipore, CB1001-500UG 1:20,000 (WB)<br>Tau12 Mouse Abcam, ab74137 Millipore, MAB2241 1:20,000 (WB)<br>CTSV - rabbit Abcam#ab166894, 1:1000 (WB)<br>DAB2 - rabbit Abcam#ab33441, 1:1000 (WB)<br>HOXC4 - rabbit Abcam#ab76093, 1:1000 (WB)<br>AUH - rabbit Abcam#ab157453, 1:1000 (WB)<br>PFKFB2 - rabbit Abcam#ab234865, 1:1000 (WB)<br>KLF11 - mouse Novus Biologicals#H00008462-M03, 1:1000 (WB)<br>STYXL1 - mouse Novus Biologicals#H00051657-B02P, 1:1000 (WB)<br>ACSM5 - mouse Novus Biologicals#NBP2-01874, 1:1000 (WB)<br>STK32B - rabbit Novus Biologicals#NBP1-32343, 1:1000 (WB)<br>PEX1 - rabbit Thermo Scientific#13669-1-AP, 1:1000 (WB)<br>Beta-actin - Mouse Abcam#ab8226, 1:10,000 (WB)
Validation	Many of these antibodies had never been tested before, and we will be the first to validate them. We tested all of them in Western blot and immunocytochemistry (IF) assays. Dilution factors listed in methods section.

Eukaryotic cell lines

Policy information about cell lines

Cell line source(s)	sAD2.1; Coriell # GM24666, (iPSCs derived from sporadic Alzheimer's disease patient; 83-year-old male), control line: Axol Bio # AX0018 (iPSC-Derived Neural Stem Cells; 74-year-old male). (SH-SY5Y (ATCC® CRL-2266)
Authentication	Cell lines were authenticated by manufacturing companies, Coriell, AxolBio, and ATCC.
Mycoplasma contamination	All cell lines tested negative for mycoplasma contamination using the Lonza MycoAlertTM Mycoplasma Detection Kit, Catalog #: LT07-118
Commonly misidentified lines<br>(See ICLAC register)	*Name any commonly misidentified cell lines used in the study and provide a rationale for their use.*
